# Regulatory RNAs and chromatin modification in dosage compensation: A continuous path from flies to humans?

**DOI:** 10.1186/1477-7827-6-12

**Published:** 2008-03-20

**Authors:** Roxani Angelopoulou, Giagkos Lavranos, Panagiota Manolakou

**Affiliations:** 1 Department of Histology-Embryology, Medical School, Athens University, Greece

## Abstract

Chromosomal sex determination is a widely distributed strategy in nature. In the most classic scenario, one sex is characterized by a homologue pair of sex chromosomes, while the other includes two morphologically and functionally distinct gonosomes. In mammalian diploid cells, the female is characterized by the presence of two identical X chromosomes, while the male features an XY pair, with the Y bearing the major genetic determinant of sex, i.e. the SRY gene. In other species, such as the fruitfly, sex is determined by the ratio of autosomes to X chromosomes. Regardless of the exact mechanism, however, all these animals would exhibit a sex-specific gene expression inequality, due to the different number of X chromosomes, a phenomenon inhibited by a series of genetic and epigenetic regulatory events described as "dosage compensation". Since adequate available data is currently restricted to worms, flies and mammals, while for other groups of animals, such as reptiles, fish and birds it is very limited, it is not yet clear whether this is an evolutionary conserved mechanism. However certain striking similarities have already been observed among evolutionary distant species, such as Drosophila melanogaster and Mus musculus. These mainly refer to a) the need for a counting mechanism, to determine the chromosomal content of the cell, i.e. the ratio of autosomes to gonosomes (a process well understood in flies, but still hypothesized in mammals), b) the implication of non-translated, sex-specific, regulatory RNAs (roX and Xist, respectively) as key elements in this process and the location of similar mediators in the Z chromosome of chicken c) the inclusion of a chromatin modification epigenetic final step, which ensures that gene expression remains stably regulated throughout the affected area of the gonosome. This review summarizes these points and proposes a possible role for comparative genetics, as they seem to constitute proof of maintained cell economy (by using the same basic regulatory elements in various different scenarios) throughout numerous centuries of evolutionary history.

## Background

The emergence and consequent prevalence of heterogamety as the dominant reproduction mechanism among the eukaryotes, was soon followed by the appearance of a genetic inequality issue that demanded to be dealt with. As is now well established, the set of sex chromosomes derived from an autosomal pair that has undergone a lengthy evolutionary process of limited recombination, degeneration and loss of gene function regarding one of the two chromosomes of the initial set. This process resulted in the formation of a derivative, largely deficient chromosome, usually denoted as Y (due to its shape in mammals), contrary to its previous homologue, now known as X. While it seems inevitable, therefore, that all genes located on the differentiated X chromosome and missing on the Y will have a two-fold level difference between the two sexes, the fact remains that most of them are not related to aspects of sexual dimorphism and hence a difference in gene product levels would actually result in a significant liability.

Dosage compensation is the term used to describe the process of attempting to equalize the gene expression between the different sets of sex chromosomes and seems to become an increasingly interesting field of research. There are many intriguing aspects to the phenomenon of dosage compensation, not the least of them the notion that several unrelated organisms seem to have independently devised similar mechanisms to achieve a certain kind of global chromosome activity regulation that would not interfere with the finely attuned control of individual genes' expression. Though these mechanisms themselves seem to differ at a first glance, a persistent repetition of elements, such as the implication of regulatory RNA molecules, the effects on chromatin structure and their strong dependence on autosomal versus X chromosome counting elements (depending on the species, either already proven or still hypothesized), require that we shed more light in what lies further down the road.

## Dosage compensation in *Drosophila melanogaster*

In the case of the common fruitfly, *Drosophila melanogaster*, like several other species, sex is determined by the presence of two X chromosomes versus a pair of X and Y. However, whereas the Y chromosome contains very few genes, mostly associated with male fertility, its counterpart houses several different genes, that are essential for a variety of functions. As a result, the process of dosage compensation in *Drosophila melanogaster *appears to have evolved, in fact, as an indispensable necessity for the continued development and survival of the species. This is accomplished by practically doubling the expression of every gene located on the single X chromosome in males, through the assembly and association of an RNA and protein complex, also known as the DCC (Dosage Compensation Complex) or compensasome (the latter term is emphasizing the complex's combined action as a cellular multi-molecular micro-machine, similar to other examples, including the apoptosome, inflammasome and proteasome) [1].

**Figure 1:**
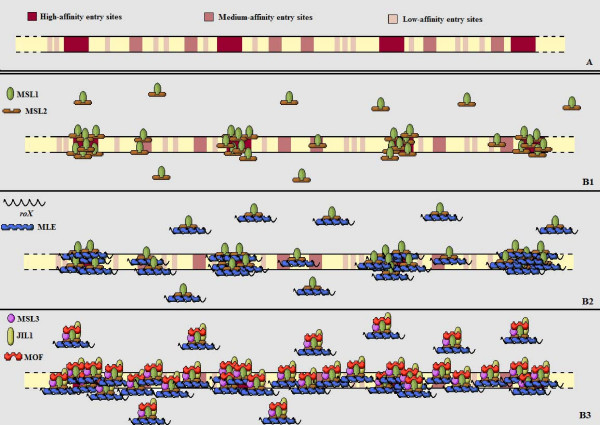
**(A) **More recent findings imply that there are in fact several different classes of chromatin entry sites with varying affinity towards the DCC. **(B1) **When only the core proteins MSL1 and MSL2 are present, they are merely capable of weakly interacting with each other and gathering around the ~35 sites that have been denoted as high-affinity entry sites. **(B2) **The addition of MLE and one of the two *roX *RNAs amplifies this interaction, but only the fully assembled complex **(B3) **can spread along the entire X chromosome by interacting with the rest of the entry sites in normal concentrations.

The six protein components of the DCC, which include the *mle *(maleless) [2,3], *msl-1 *(male-specific lethal) [4], *msl-2 *[5], *msl-3*, *mof *(males absent on the first) [3] and *jil-1 *products, when combined with the non-coding RNAs *roX1 *and *roX2 *(RNA on the X) [6-8] form an active complex which is localized on the X chromosome. Complex formation is rapidly followed by enrichment with H4Ac16, a specific histone isoform that has been acetylated at lysine 16 and which leads to a change in chromatin structure. Thus, the ultimate result of compensasome constitution is an increased transcriptional activity of every gene located on the single male X chromosome (opposite to mammalian X inactivation, described later) [9-11] [see Figure 1].

Sex specific regulation of this mechanism is achieved by the fact that all components of the DCC must be present to allow it to bind and expand across the entire X chromosome. And while this has been validated for all six proteins and the *roX *RNAs, it is *msl-2 *especially, and, to a lesser degree, *msl-1*, that suppresses this mechanism in females, since it has been established that the sex determining switch protein SXL, which is only present in females, deregulates translation of *msl-2 *transcripts [12,13].

Once male status has been established through *sxl *and consequently *msl-2*, a complex mechanism that encompasses the assembly of the DCC and its binding to the X chromosome is initiated. The core of the DCC predominantly consists of the MSL1 and MSL2 proteins, which bind together and exhibit a weak interaction with circa 35 sites across the X chromosome. The latter are described in the literature as chromatin entry sites. Two of these entry sites correspond to the *roX1 *and *roX2 *genes as well [6,7]. Interestingly, the MLE protein, which is expressed in both sexes, but only appears to participate in this process in males, is necessary for the MSL1 and MSL2 proteins to further interact with the *roX *RNAs [6,7]. Consequently, it is assumed that MLE stabilizes the original complex and its binding to the initial chromatin entry sites.

To complete compensasome activity in *D. melanogaster*, the recruitment of additional proteins is necessary, since a fully functional dosage compensation complex requires the integration of the remaining three proteins previously mentioned, i.e. MSL3, MOF and JIL1 kinase. MSL3 has been known to be able to interact with chromatin, whereas MOF displays H4 histone acetyltransferase activity [10]. Once the DCC has been fully assembled, it gains the ability to interact with several more sites and can consequently be located (expanded) across the entire X chromosome.

The missing details of the mechanism that allows targeting the single X chromosome among the autosomes have been a hot topic of intensive research recently. It was initially believed that the DCC could spread along *in cis*, initially interacting with chromatin around the 35–40 chromatin entry sites and thus, gradually covering the entire chromosome [14,15]. This was supported by data reporting that integration of a *roX1 *or *roX2 *gene, which contains a chromatin entry site, to an autosome led to hyper-transcription of neighboring autosomal genes, but this adaptive reaction was limited only in the immediately proximal area of the chromosome [16,17].

However, it was soon also discovered that inserting a part of the X chromosome without a major entry site would still attract the compensasome and result in epigenetic chromatin modification, namely acetylation and hypertranscription. On the other hand, inserting a part of an autosome into the X chromosome would usually display dosage compensation effects at its connecting points, but not the complete spreading that is normally observed along the rest of the X chromosome [18].

Newly presented data has suggested that, instead of the 35–40 major chromatin entry sites alone, there may in fact exist a wide population of chromatin entry sites with varying affinity for the DCC [18-21]. According to this theory, while high-affinity sites may suffice for the initiation of the DCC *in-cis *spreading on their own, medium and low level affinity sites may need to interact with several mediators or even in combinations, to be successful as additional binding positions for the DCC [19]. What now remains to be seen is what exactly these varying entry sites consist of.

Besides targeting, further questions concerning dosage compensation in the fruitfly still remain to be answered. While it is true that, through the control of *sxl*, the absence of MSL2 alone is responsible for the inhibition of the DCC assembly in females, the rest of the compensasome components seem to be present in females as well, displaying a uniform low level association with all chromosomes [22]. The hypothesis that dosage compensation in *Drosophila *doesn't simply concern X chromosome hypertranslation, rather it may affect autosomal expression as well under various alterations, also seems intriguing. For example, achieving DCC assembly and targeting the pair of X chromosomes in females does not result in their hyper-transcription, but rather in autosomal gene down-regulation [23]. Furthermore, the majority of authors agree that histone acetylation of the X chromosome alone does not seem to be sufficient to account for all attributes of this phenomenon.

In addition, it has become apparent through more recent studies that dosage compensation in *Drosophila *is a far more dynamic mechanism than we may have imagined. It seems, for example, that DCC binding can differ between different cell types, with a distinct predilection for targeting transcriptionally active genes [24]. Furthermore, this profile of DCC binding appears to be established during early stages of development and afterwards, it shows only minor differences, despite the extensive changes observed in gene expression, promoting the hypothesis of an early establishment of DCC binding [25]. The mechanism of this interaction within the context of dosage compensation has yet to be established, although recent data point towards the existence of target sequences within the coding regions of many genes that are only revealed in the presence of the transcription machinery [26].

Finally, and more importantly, one cannot ignore the fact that there are genes within the X chromosome, such as *Lsp-1α*, which have homologues on the autosomes [27], or the female-specific yolk protein genes [28], which seem to escape the effects of dosage compensation. Others, such as the *runt *gene, are dosage compensated post-transcriptionally by SXL in females [29]. One theory attempting to explain this controversial discovery postulated that this may be the result of quantitative differences in the levels of *in trans *acting autosomal transcription factors [22]. The fact remains, however, that more light needs to be shed on if and how this may be achieved.

## Dosage compensation in other species: Many questions, no answers yet

Dosage compensation is thought to be a mechanism that may, theoretically, be necessary for all species exhibiting sexual dimorphism at a chromosomal level, since this mechanism includes a differentiated expression of a certain number of genes in each sex. Evidently, the understanding of this procedure is directly associated with the detailed description of the pathway of sex determination and differentiation in the various species and, indeed, progress in dosage compensation basic research has only been observed in cases where sexual choice had been already adequately understood and described at a molecular level.

Advances in sex determination genetics, comparative biology and reproductive physiology of reproduction have revealed that chromosomal sex determination is a very common process in nature, extending to various animals of diverse evolutionary background, such as birds, fish, amphibians, reptiles and, of course, mammals. In all the above cases a pair of chromosomes is considered to be the major determinant of sex, usually referred as gonosomes or sex chromosomes. Should this pair be identical in the female and differentiated in the male, the terminology XX, XY is applied, deriving from the macroscopic description of these chromosomes in eutherian mammals. If the opposite is true, as is most common in avian animals/birds, the nomenclature changes to ZZ, ZW, to apply to the closest available letters in the English alphabet. Indeed, this choice was meant to stress the evident analogues between these two major chromosomal sex determination strategies and their potential common evolutionary history. Although this issue remains debatable to this date, with various researchers presenting arguments in favor or against this generalized unifying theory, recent data appears to suggest a common background for XY and ZW, with episodes of genomic transition among them [30-33]. In particular, it seems that there is a degradation process for both Y and W and a significant inter-species conservation rate for both X and Z. Although X and Z structures and genomic sequences are completely different, their presence in closely related species (e.g. fish) in highly conserved form and the knowledge that single gene sexual dimorphism has been detected in some organisms of the same group leads to the hypothesis that there might indeed be a common ancestry for all sex chromosomes [30].

Regardless of the answer to whether a common origin for all gonosomes may indeed have existed, it is true that the necessity for dosage compensation has so far been proven for all animals exhibiting chromosomal sex-specific variation and submitted to full genomic analysis, e.g. nematodes, flies and mammals. Therefore, it is reasonable to assume that a similar process might be present in other animals with an XX/XY or ZZ/ZW chromosomal sex determination system, which could also generate genetic dose inequality between the sexes. However, this may be examined only if a full genomic analysis is available for these species, a process which may require a few extra years. This is, for example, the case with reptiles, the genomic analysis of which has only recently been proposed as a fully operational project [31]. Therefore, until data becomes available for these species as well, the existence of dosage compensation in animals other than the model organisms already examined in detail (basically *Caenorhabditis elegans*, *Drosophila melanogaster *and *Mus musculus*) largely remains a supposition and is, therefore, impossible to discuss further, at present, in the context of a review paper.

A few recent publications have enriched the literature with insight as to the presence of dosage compensation in other animals as well. In particular, studies performed in chicken and zebra finch demonstrate that dosage compensation must occur to some extend in these species as well, but it doesn't achieve the equality in gene expression observed in other animals with a chromosomal sex determination pattern [33-35]. This is most surprising, since, even in the case of humans, gene expression in males and females is comparable for both autosomes and gonosomes, despite the existence of genes that escape X chromosome inactivation, contrary to birds, where the expression rate is profoundly different in the two above-mentioned cases [35-38]. The mechanism of dosage compensation in birds, itself, remains unknown, with several candidate players (e.g. non-coding RNAs, similar to *rox*) suggested, but no actual proof concerning their potential role in the process reported in the literature so far [33-35].

## Dosage compensation in mammals: X chromosome inactivation

In mammals, sex is determined by the presence of the Y chromosome and, more specifically, its male specific region (MSY), a non-recombining region (apart from self recombining repeats within the chromosome) organized around the major sex determinant gene. i.e. SRY (sex determining region of the Y chromosome). Based on comparative genetic analysis, scientists have shown that the SRY is common to all mammals and, most probably, constitutes the initial event in a series of successive phenomena (recombination failure, sequence duplication, genomic transfer, translocation) that led to gradual X-Y distinction, from a pair of identical autosomes to their current form. As a result of these genomic variations, the Y chromosome has gradually deteriorated, maintaining only minimal homology to the X chromosome, mainly detected in the two distant arms of the chromosomes, which constitute the so called pseudo-autosomal regions 1 and 2 (symbolized as PAR1 and PAR2, respectively) [36-38]. On the other hand, this gradual deterioration has lead to a considerable excess of genetic information in XX females, bearing two X chromosomes, compared to XY males, with a single copy. The means to overcome this inequality has been developed early in mammalian evolution in the form of the effective transcriptional silencing of almost the whole of all but one X chromosomes, in all diploid cells of marsupial and eutherian mammals. This process has been originally described by Mary Lyon in mice and is now known as "the Mary Lyon hypothesis of X chromosome inactivation" or simply "Lyonization hypothesis," a principle that continues to be accepted with minimal reforms after almost 50 years from its initial publication [38-40].

In the original version, the Lyon hypothesis referred to X chromosome inactivation in mice, proposing the following basic characteristics:

1. X chromosome inactivation is catholic, in the sense that it refers to the translational silencing of all the content of one X chromosome in every diploid cell of a typical 46, XX female, with no exception. In subsequent years, this concept was further supported by evidence that the inactive X chromosome actually underwent a rapid transformation to structural heterochromatin (Barr Body).

2. X chromosome inactivation is random in somatic tissues, i.e. in each diploid cell of normal females either the paternal or the maternal copy will be inactivated. However, this choice is repeated in all derivative cells in a constant manner, i.e. the inactivation motif is retained in a clonal way. This, effectively, justifies the characterization of females as functional mosaics of X-linked genes, in case the information coded in the alleles of the same locus in each X chromosome is different.

3. X chromosome inactivation occurs very early in development. Indeed, the period of initiation of X inactivation has been proposed to correspond to the end of the first week of gestation, which proves its necessity for normal embryonic growth and subsequent tissue differentiation and commitment to specific cell lineages.

In the years that followed the followed the initial description of the Lyon hypothesis, a number of researchers enriched the literature with information on the molecular mechanism of X chromosome inactivation and its variations among different mammalian species. Thus, we now know, that some genes on the X chromosome escape/evade inactivation and, therefore, this process is not truly complete, as Mary Lyon believed. At least 35 such escapees have been described so far in the human X chromosome and their distribution appears to be non-random, although its exact significance hasn't been understood yet. Moreover, despite the initial belief that X chromosome inactivation is by definition a random selection phenomenon, subsequent research has revealed that this is only partially true [36-40].

For instance, in monotremes, the non homologous section of the gonosomes is restricted to the area around the SRY gene and, thus, only this section is submitted to inactivation/transcriptional down-regulation. In marsupials, X chromosome inactivation is a more generalized phenomenon, but the choice of the X to be inactivated is not random [40]. In fact, it seems to follow an epigenetic imprinting principle, according to which, the X of paternal origin is the one marked as the target of the inactivation mechanism, while the maternal one remains fully active. Naturally, this operation limits the capacity to promote genetic variation, since the ability to co-express different alleles in a mosaic fashion is only available in random-selection systems, as is the case in eutherian mammals. More interestingly, in some of the latter, such as rodents (mice), X chromosome inactivation appears to maintain a transient pattern, with somatic tissues of the embryo featuring random choice of the inactive X, while the extraembryonic tissues continue to demonstrate an imprinted pattern [38].

In an attempt to explain this paradox, most scientists seem to conclude that X chromosome inactivation is an adaptive mechanism that has developed gradually over centuries of evolutionary history, initially using basic molecular tools. The latter were gradually enriched and updated to more sophisticated and complex patterns of interaction. If this is indeed accurate (and the study of various mammals along their evolutionary tree, focusing on X-Y structure and dosage compensation pattern, appears to validate this concept so far) it would imply that X chromosome inactivation appeared as an adaptive response to the increasing need for a countermeasure to gene inequality among X and the deteriorating Y chromosome. This process may have initiated using basic translational control means ubiquitously available, i.e. functional non-coding RNAs (ribozyme equivalents) and histone modification epigenetic regulatory elements (mostly acetylation and methylation). Indeed, the preservation of these elements in major regulatory/adaptive reactions (including dosage compensation itself) in a variety of evolutionary distinct species appears to validate this theory. Non-coding RNAs, for instance, are present in birds, although their role is yet unclear. In humans, they are also involved in genomic imprinting, as has been revealed by the study of relevant diseases, such as the Prader-Willi syndrome, Beckwieth-Wiedermann syndrome and Mc Cune-Albright syndrome.

Within the context of cell economy, the selection mechanism (i.e. which X will be silenced) may also be associated with these common cellular regulatory tools. The role of non-coding RNAs, such as *Tsix*, is clear in the case of mice, where it appears to influence the production and availability of the major regulator of X inactivation, i.e. *Xist*. In the extra-embryonic tissues, the process is also operating via methylation imprinting of X-linked sites, such as the DXPas locus. The development of a more advanced, random selection model is viewed in this approach as a later evolutionary event and, for this reason, it is only partially observed in rodents and it has completely removed imprinting-based selection only in primates and humans, where the equivalent of *Tsix*, i.e. *TSIX*, has no reported function [37-41].

Still, even in the latter, random choice is not an infallible process and several cases of non random selection have been described, with a possible link to the onset of various diseases. This is known as the skewing principle for X chromosome inactivation or skewed X inactivation and it remains a debatable issue both in terms of etiology (disruption of X chromosome integrity is only detected in a minority of subjects) as well as succinct evaluation of its clinical significance (if any) [38-42].

## X chromosome inactivation pathway: A three step silencing operation

Although, as previously described, X chromosome inactivation is common to all mammals, it has been described in greater detail in mice, where it was also initially discovered. Subsequent studies in other mammals are, thus, mainly aiming to examine the presence and role of various mediators already known to participate in the process in rodents. For this reason, the model further described is completely accurate only for mice, while additional specific references are also made to humans wherever relevant data is available.

X chromosome inactivation is a process that rapidly results in the loss of expression of almost all genes located on the targeted chromosome (indeed, minimal expression of genes is detected prior to inactivation on both X chromosomes, including, most interestingly, genes directly associated with the inactivation process itself, such as Xist). This observation implies that there must be an initial phase when the number of autosomes and gonosomes is somehow counted and the choice of the X to be inactivated is made (based on either imprinting or random selection). Nevertheless, it is also a stable process, because, once established, it is retained in a most reliable manner in all future tissues. Therefore, a molecular model must explain a)how inactivation commences early during fetal development b)how this initial signal rapidly spreads along the whole X chromosome (but also evading some specific sites, as previously reported) and c)how this mechanism is stabilized, so that it can securely and accurately extend to all derivative cells. These three distinct phenomena are usually described as the three stages of X chromosome inactivation, i.e. initiation (including chromosome counting and inactive X selection), expansion (including inactivation message expansion, chromosome coating and gene silencing) and maintenance (epigenetic regulation/chromatin modification aiming to maintain the inactive state) [41,42].

The initiation stage is characterized by the selection of the X chromosome to be inactivated. Although the exact mechanism remains to be determined, it seems that the process is based on the creation of an initiation complex, which includes a blocking factor (BF) coded by one or more autosomes (thus serving as an indicator of autosomal chromosome number in the cell) and a binding site for it on chromosome X. The latter area is an indirect means to determine the number of X chromosomes and is therefore described as the counting element (CE). In organisms with random X chromosome inactivation, the creation of the initiation complex is viewed as a competitive phenomenon, where the quantity of BF in diploid cells is always enough to bind to all available CEs but one. In this case, in normal XX females one X is targeted for inactivation while in normal XY males none. As was previously mentioned, neither the BF nor the CE have been accurately detected in any mammal to this date and, therefore, the initiation complex theory remains to be verified [43]. A number of more complicated events, such as the homologous pairing of X chromosomes may be also significant in the initiation of this process.

**Figure 2:**
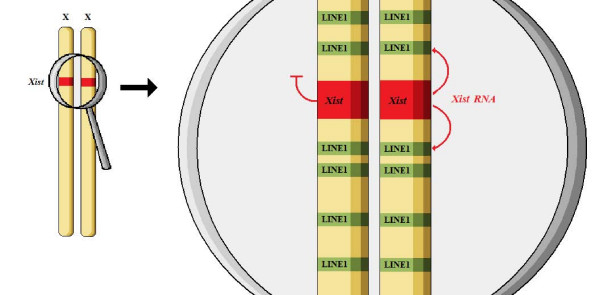
The initiation stage of XCI.

The first signal of a commitment towards X chromosome inactivation is the stable expression of *Xist *(X inactive chromosome specific transcript). This gene is located on the X chromosome, in the section which includes most X inactivation-associated regulatory elements usually described as X inactivation center. The gene produces a non-translated functional RNA, which is only temporarily expressed in pre-inactivation X chromosomes, while its stable persistent expression characterizes the inactive X only [see Figure 2]. This is an interesting exception to the general rule by which the opposite is usually true, i.e. the largest number of X-linked genes are only active on the non-inactivated X. Since the period of *Xist *parallel expression on all Xs is only minimal, it is considered as a marker of X inactivation widely used in the literature. From a mechanistic point of view, it appears that the BF-CE complex, if indeed present, must somehow hinder the further presence of *Xist *RNA by acting at the promoter region or other transcription-associated regulatory elements [43-46].

In animals with non-random X inactivation, a number of additional factors are involved in X selection [47]. For example, in mice, such loci include the Xce and Xite regions, also known as choice elements. If inactivation follows an imprinted pattern, as is the case in marsupial mammals or extraembryonic rodent cells, then there must also be a methylation variation at the epigenetic level [47], occurring either around the Xist locus or around the *Tsix*, an area (XPas 34) that produces a complementary antisense transcript thought to directly or indirectly hinder *Xist *action in mice. On the other hand, it should be noted that data available for human X inactivation is more limited, but it verifies the presence of both *Xist *and *Tsix *analogues, of which the first retains a crucial function, while the possible contribution of the second is still a controversial issue. No other imprinting or choice elements are known in humans, acting either in *cis *or in *trans *[48-50].

**Figure 3:**
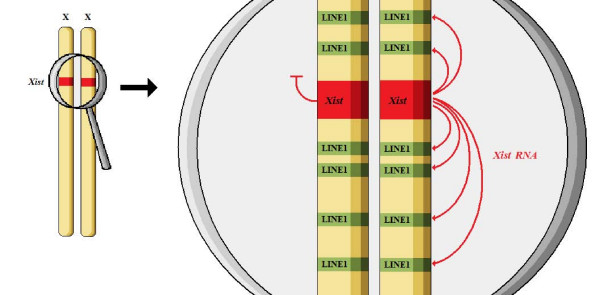
The expansion stage of XCI.

The second or expansion stage of X inactivation is characterized by the rapid coating of the X chromosome chosen to be inactivated by *Xist *RNA. This phenomenon is described as spreading reaction and covers all the X, apart from the two distant pseudoautosomal regions. It is based on a gradual expansion principle, according to which, *Xist *RNA binds to specific regions distributed non-symmetrically along the X chromosome. These regions are evolutionary conserved, non-coding sequences (Line1 sequences) and they may be a direct target for *Xist *RNA or part of a larger complex, also involving protein interaction, that remains to be detected [46]. The coating process results in effective initial genetic silencing, excluding only few genes along the chromosome that do not seem to demand dosage compensation [see Figure 3]. A comparative presentation of the basic features of both *roX *and *Xist *RNAs is provided in Table 1.

**Table 1:** Non-coding RNAs in dosage compensation

**Feature**	***roX RNA***	***Xist RNA***
Number of genes	2	1
Site of production	X chromosome	X chromosome
Length	3.7 kb (*roX1*) 0.5–1.2 kb (*roX2*)	19.3 Kb
Expression duration	Retained over time	Transient
Sex specific expression	Yes	Yes
Role in compensasome-condensasome formation	Yes	Possible
Necessity	Initiation, Maintenance	Initiation, Spreading
Expansion *in cis*	Limited	Along the chromosome
Expansion *in trans*	Yes	Yes
Coating/Spreading	Limited (debatable)	Definite, large scale

Table summarizing the key features of the two major types of non-coding RNAs involved with dosage compensation.

**Figure 4:**
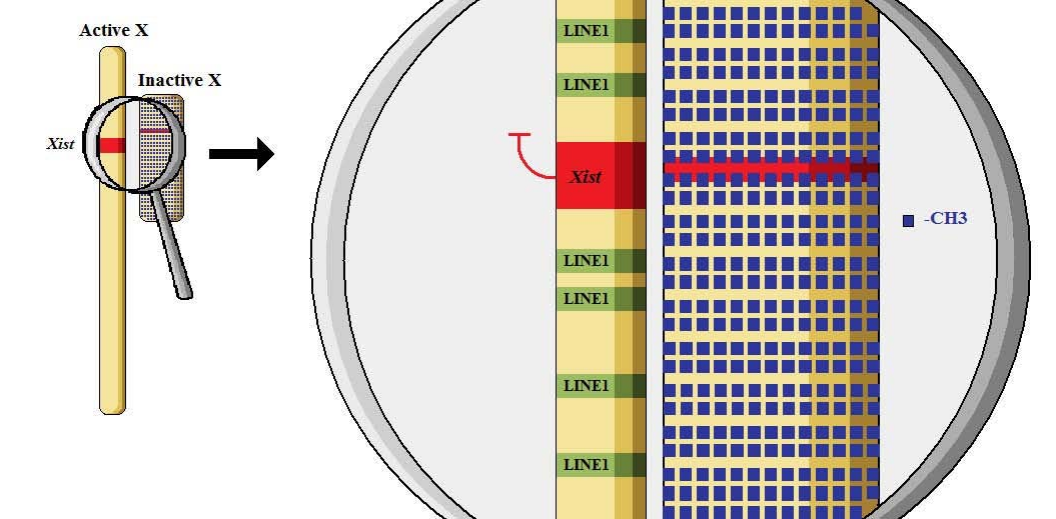
The maintenance stage of XCI.

The final inhibition of further gene expression and the structural transition towards heterochromatin formation (Barr body creation) is the object of the third and last stage in X inactivation, the maintenance stage [see Figure 4]. This is an almost exclusive epigenetic regulation phase, involving extensive DNA methylation in CPG islets, as well as histone hypermethylation and hypoacetylation (Lysine). Finally, macrohistone formation is also a common observation [51-54]. In the process of maintenance of X inactivation in mice, several other genes have been proven to be major players, including members of the Polycomb group (e.g. *eed*), the product of which is a mediator participating in histone methyltion and deacetylation. All the above interactions result in a differentiated biochemical profile and three dimensional space distribution, which makes the interaction of the inactive X chromosome with RNA polymerases almost impossible, thus establishing a stable and complete genetic silencing pattern. The use of chromatin modification strategies to achieve massive gene expression regulation is a classic motif in nature, also observed in a variety of species and in different normal and pathological scenarios [55-58] and details for its use in dosage compensation may be found in Table 2.

**Table 2:** Epigenetic phenomena in dosage compensation

***Feature***	***Drosophila***	**Mammals**
Histone Acetylation	H4Ac16, special histone isoform acetylation	Hypoacetylation
CpG Methylation	Not described to date	Hypermethylation
Histone Methylation	Not described to date	Hypermethylation
Histone Phosphorylation	Assumed (kinase action)	Not described to date
Macrohistone Formation	Not described to date	Yes
Selection Pattern	No need for selection	Imprinted/Random

Comparison of epigenetic phenomena involved in dosage compensation in the fruitfly and mammals.

## Comparing the RNAs

Having reviewed the major points of dosage compensation in two largely distant species, *Drosophila melanogaster *and humans, one significant detail seems to immediately draw attention: the implication of non-translatable RNAs as a means to target the appropriate chromosomes [59-63].

**Figure 5:**
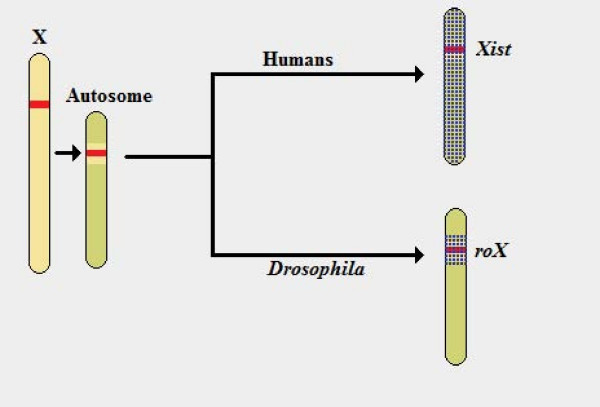
While both *roX *and *Xist *retain the ability to initiate dosage compensation when transferred to an autosome, *Xist *has the potency to spread along the entire chromosome, whereas *roX *has only been known to affect neighboring genes. However, even in the case of *Xist *RNA, the ability to spread along an entire chromosome depends on both the number of gene copies and the presence of an adequate number of booster elements (i.e. binding sequences, such as Line 1).

Indeed, both *roX *and *Xist *RNAs exhibit a unique function as far as RNA molecules are concerned, being exclusively associated with the field of dosage compensation [62-64]. It also seems interesting to note that, although it is unlikely that these two may share a common ancestor RNA molecule performing the same role, since the two species were separated before the emergence of heterogamety and even their sex chromosomes derive from different sets of autosomes, they exhibit some intriguing similarities. To begin with, they are both excellent examples of RNA molecules that are submitted to the processes of splicing and polyadenylation, when neither of them is allowed to exit the cell nucleus or has an identifiable reading frame that may allow their translation into a protein molecule. They are also both the only RNA molecules that have been described to date as being capable of interacting with and spreading across the chromosome that houses their gene locus. Moreover, this ability is retained even when the relevant gene is inserted in another chromosome, in which case they exhibit the same behavior by interacting with the new chromosome, limited as that may be in the case of *roX*, or even *Xist*, if Line-1 binding sequences are missing [see Figure 5]. In addition, both RNAs are apparently capable of specifically exempting certain genes from the delicate chromosome silencing process [64-66].

However, there are also important differences that one cannot justifiably disregard. For instance, although both RNAs bind to active chromosomes, *Xist *RNA results in its transcriptional suppression (i.e. inhibition of gene expression – gene silencing) while, on the other hand, *roX *RNA permits gene expression. Mammalian *Xist *RNA can only act *in cis*, since the entire process of X inactivation is meant to be engaged in only one of the two X chromosomes. Allowing the *Xist *the ability to act *in trans *would have resulted in loss of control over the entire selection process. On the other hand, the *roX *RNAs have been long known to be capable of both *in trans *accumulation and *in cis *spreading with the aid of the dosage compensation complex and so long as the appropriate entry sites are available. Whether that is a result of having to ensure that the entirety of the single X chromosome will be properly involved or a mere by-product of the fact that there is no need to ensure the process will only be constrained in one of two X chromosomes remains unsure. Besides, one cannot forget that, due to their very own nature, the two *roX *RNA were only meant to spread from the circa 35 major entry sites across a mere total of 25 Mb that consist the *Drosophila*'s X chromosome, whereas human *Xist *RNA has to spread across an area of more than 100 Mb from the single location of its production site [64-66].

## Common elements, common strategies: Perhaps common history as well?

The description of the basic dosage compensation strategies in this review has emphasized some key similarities between the two model organisms mostly analyzed, namely the common fruitfly and the mouse [see Table 3]. These may of course be attributed to chance without any further biological or even scientific interest in general. However, the use of so compatible strategies in species as evolutionary distant as these still appears to be a most remarkable phenomenon [67-69]. When one also takes into account the fact that non-coding RNAs are considered by evolutionary biology the most primitive kind of functional machinery devised in the cell (even before the large scale use of protein enzymes) and that epigenetic regulation at the level of histones is an almost universal biological principle (from all eukaryotic cells, only spermatozoa lack histones as a result of protaminosis), it may be reasonably assumed that this retained cell economy may not be simply attributed to chance. Naturally, the current lack of knowledge concerning sex determination in various species across the evolutionary tree and the even greater lack of data on dosage compensation makes the accurate development of evolutionary hypotheses, supported by solid evidence, impossible at present. However, several research projects are in progress and their findings will aid considerably this task, since the discovery of similar dosage compensation patterns in other animals, such as reptiles or birds, will support the argument of a common evolutionary history for all these species, at least with regard to sex chromosome origin [67-69].

**Table 3:** Dosage compensation mechanisms: an overview of their basic characteristics

***Feature***	**Drosophila**	**Mammals**
Gene regulation result	Hypertranscription	Transcriptional silencing
Genes escaping effect	Different cases already established	Established (humans/mice)
Clonality	Assumed	Established
Outcome in case of Quantitative Chromosomal Abnormalities	Sex reversal or lethality (sex determined by the ratio of autosomes to gonosomes)	All but one X are inactivated in diploid cells (X-1 rule)

Comparison of the basic characteristics of dosage compensation in flies and mammals.

## Abbreviations

**DCC**: Dosage Compensation Complex; **Eed: **Extra embryonic deficient; **H4Ac16**: H4 histone acetylated at lysine 16; **Line1**: long interspersed nuclear elements 1; ***mle***: maleless; ***msl***: male-specific lethal; ***mof***: male absent on the first; ***roX***: RNA on the X; ***sxl***: sex lethal; **Tsix**: Xist backwards (antisense transcript); **XCI**: X chromosome inactivation; **Xic**: X inactivation center; **Xist**: X inactive specific transcipt

## Competing interests

The author(s) declare that they have no competing interests.

## Authors' contributions

All three authors contributed equally in manuscript preparation, proofreading and final production. All figures were developed by P. Manolakou and all tables by G. Lavranos, but their final content was discussed and reviewed by all three authors.
